# Targeted Delivery of c(RGDfk)‐Modified Liposomes to Bone Marrow Through In Vivo Hitchhiking Neutrophils for Multiple Myeloma Therapy

**DOI:** 10.1002/advs.202409895

**Published:** 2024-12-16

**Authors:** Huiwen Liu, Bo Zhang, Hongrui Chen, Honglan Wang, Xifeng Qin, Chunyan Sun, Zhiqing Pang, Yu Hu

**Affiliations:** ^1^ Institute of Hematology Union Hospital Tongji Medical College Huazhong University of Science & Technology Wuhan Hubei 430022 China; ^2^ Key lab of Molecular Biological Targeted Therapies of the Ministry of Education Union Hospital Tongji Medical College Huazhong University of Science and Technology Wuhan 430022 China; ^3^ Department of Pathology Union Hospital Tongji Medical College Huazhong University of Science and Technology Wuhan 430022 China; ^4^ School of Pharmacy Fudan University Key Laboratory of Smart Drug Delivery Ministry of Education 826 Zhangheng Road Shanghai 201203 China

**Keywords:** bone marrow targeting, c(RGDfk), liposomes, multiple myeloma, neutrophil hitchhiking

## Abstract

Multiple myeloma (MM) is a prevalent bone marrow disorder. The challenges in managing MM include selecting chemotherapy regimens that effectively modulate the myeloma microenvironment and delivering them to the bone marrow with high efficacy and minimal toxicity. Herein, a novel bone marrow targeting strategy using c(RGDfk) peptide‐modified liposomes loaded with chemotherapeutics is developed, which can specifically recognize and hitchhike neutrophils following systemic administration, capitalizing on their natural aging process to facilitate precise drug delivery to the bone marrow, thus minimizing off‐target effects. On the one hand, c(RGDfk)‐functionalized liposomes containing carfilzomib (CRLPs) successfully transformed macrophages from M2 phenotype to M1 phenotype, enhancing immunotherapeutic responses. On the other hand, c(RGDfk)‐functionalized liposomes encapsulating BMS‐202 (BRLPs), a small molecule checkpoint inhibitor, interrupted the PD‐1/PD‐L1 axis and promoted the infiltration of cytotoxic T cells. The co‐administration of CRLPs and BRLPs successfully delivered drugs to bone marrow, leading to significant modulation of the myeloma microenvironment, reduced tumor growth, and improved survival time of MM‐bearing mouse models. These findings introduced an alternative approach to modulating the myeloma microenvironment and underscored the efficacy of hitchhiking neutrophils for bone marrow drug delivery. This strategy show advantages over traditional drug delivery methods in terms of improved efficacy and lowered toxicity.

## Graphical abstract

A novel bone marrow‐targeting strategy employs c(RGDfk)‐modified liposomes to hitchhike aging neutrophils, enabling precise drug delivery and reducing off‐target effects. These neutrophils deliver BMS‐202 to block PD‐1/PD‐L1 and carfilzomib to polarize macrophages into M1‐type, transforming the tumor microenvironment into an immunogenic state and achieving effective therapy in MM‐bearing mouse models.

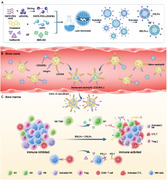

## 1 Introduction

Multiple myeloma (MM) arises from abnormal accumulation of cancerous plasma cells in the bone marrow, which is the second most common type of hematological malignancy. Currently, there is no cure for MM, and high recurrence rates are common. One of the primary reasons is the existence of a bone marrow immunosuppressive microenvironment, which is characterized by a high abundance of immunosuppressive cells like regulatory T cells (Tregs) and M2 macrophages, along with a reduced population of tumoricidal cell types like cytotoxic T cells (CTLs).^[^ ^1^ ^]^ Large amounts of clinical evidence are accumulated to demonstrate that the immunogenic status of the bone marrow immune microenvironment correlates positively with the clearance of minimal residual disease in MM, which further influences the progression‐free survival and overall survival of MM patients.^[^ ^2^, ^3^ ^]^ With the advent of new therapeutic drugs, such as proteasome inhibitors, immune modulators, and anti‐CD38 antibodies, the progression‐free survival and overall survival of MM patients are greatly improved. Despite these advancements, the recurrence of MM underscores the inability of current therapies to completely eradicate minimal residual disease associated with MM. In addition, refractory/relapsed MM (RRMM) patients have shown limited therapeutic benefits from programmed death 1 (PD‐1)/programmed death ligand 1 (PD‐L1) antibodies, despite their high efficacy in treating some solid tumors.^[^ ^4^ ^]^ To improve their efficacy, a clinical trial that combined the checkpoint inhibitors with the Food and Drug Association (FDA)‐approved first‐line immune modulator drug lenalidomide was conducted. Unfortunately, this trial was suspended due to severe side effects.^[^ ^5^ ^]^ Therefore, the development of new strategies that can safely and effectively modulate the bone marrow microenvironment is crucial for achieving a cure for MM.

In addition to selecting appropriate drugs for MM immunotherapy, delivering them to bone marrow with high efficiency is equally challenging. This is primarily due to low blood perfusion and the existence of the bone marrow blood barrier, which hinders small‐molecule drugs/nano drugs from reaching the bone marrow target after systemic injection.^[^ ^6^ ^]^ Therefore, increasing therapeutic doses has been a conventional strategy to enhance efficacy, but it often leads to elevated toxicity and side effects. Besides, various strategies have been proposed to directly target the bone marrow using aptamers, peptides, or antibody‐modified nanodrugs, but the improvement of the delivery efficacy remains a critical issue.^[^ ^7^ ^]^

**Scheme 1:**
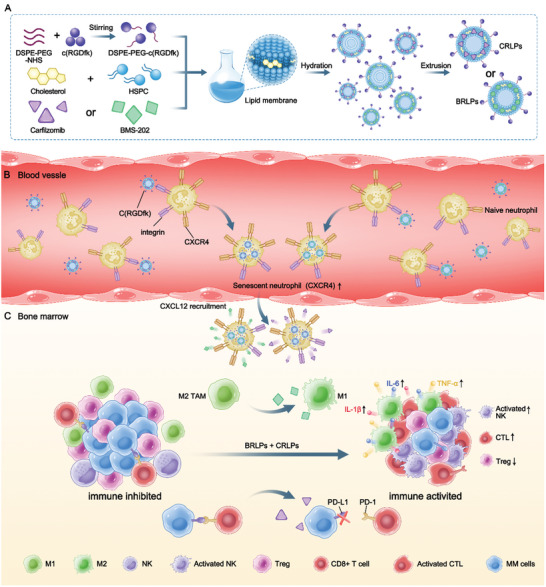
(A) The preparation process of BRLPs and CRLPs. (B) BRLPs and CRLPs hitchhiked on neutrophils in vivo by binding to integrin receptors, exploiting them as carriers to migrate to the bone marrow during neutrophil aging. (C) The bone marrow homing neutrophils released BMS‐202 which interrupted the PD‐1/PD‐L1 axis and carfilzomib (CFZ) which induced macrophage polarization into M1‐type, leading to reinvigoration of tumor microenvironment from immunosuppressive into immunogenic status.

Neutrophils constitute the primary element of the innate immune system, which serves to protect against infections. For neutrophils that do not migrate into pathological tissues, aging is their final destiny, which is characterized by upregulated expression of C‐X‐C chemokine receptor type 4 (CXCR4), a receptor that facilitates their homing to the bone marrow.^[^ ^8^ ^]^ Inspired by this naturally occurring phenomenon, here we developed a novel liposomal drug carrier modified with c(RGDfk) peptide (RLPs), which could specifically hijack neutrophils in vivo, be transported to the bone marrow after the neutrophils age, and release the therapeutic payloads there (**Scheme** **1**). Using the in vivo MM‐bearing mice models, we demonstrated that the combination of RLPs loaded with BMS‐202, a small‐molecule checkpoint inhibitor (BRLPs), and RLPs loaded with carfilzomib (CFZ), a proteasome inhibitor (CRLPs), significantly inhibited tumor growth and improved survival. Mechanism studies indicated a decrease in the percentages of immune‐inhibitory cells, including CD206^+^ M2 type macrophages and Tregs, alongside an increase in the percentages of CTLs and natural killer (NK) cells within the local microenvironment of bone marrow after systemic administration of CRLPs and BRLPs. Our data underscored the potential of hitchhiking neutrophils in vivo as a promising strategy for delivering therapeutic payloads to bone marrow and providing an alternative combined therapy for effective MM treatment via myeloma microenvironment modulation.

## 2 Results and Discussion

### 2.1 The Characterizations of RLPs

**Figure 1:**
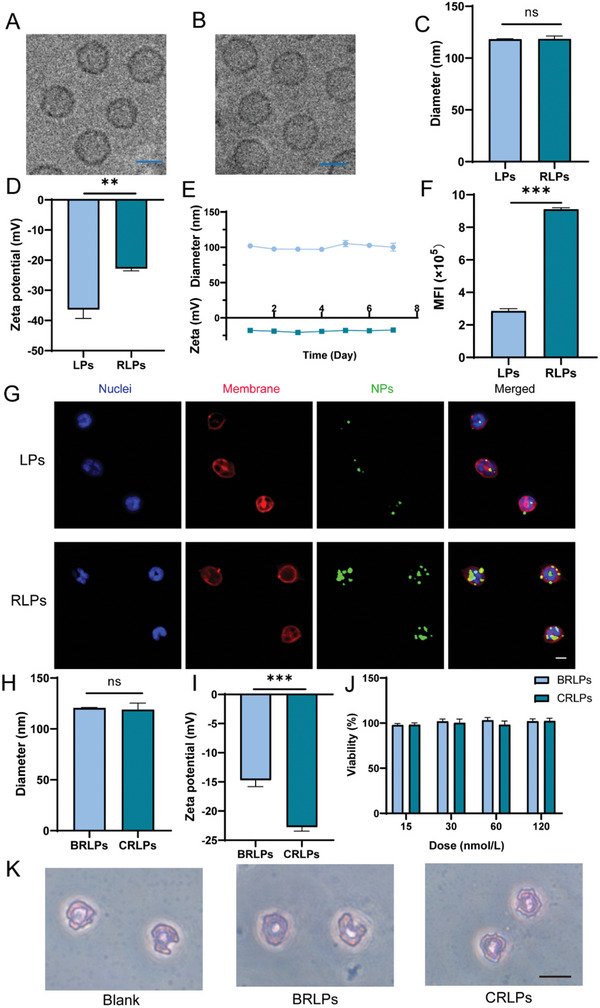
The characterizations of RLPs. Cryo‐TEM images of LPs (A) and RLPs (B). Scale bar, 100 nm. (C,D) The diameters and zeta potential of LPs and RLPs. (E) The stability of RLPs is indicated by size and zeta potential in deionized water at 4 °C for a week. (F) The mean fluorescence intensity (MFI) of neutrophils detected by flow cytometry after incubation with DiD‐labeled LPs and RLPs for 1 h. (G) The CLSM imaging of neutrophils after incubation with LPs and RLPs, respectively. Blue, DAPI‐stained nuclei. Red, DiI‐labeled cell membranes. Green, DiD‐labeled LPs or RLPs. Scale bar, 10 µm. (H, I) The diameters and zeta potential of BRLPs and CRLPs. (J) The cell viability of neutrophils after incubating with various concentrations of BRLPs and CRLPs for 1 h (*n* = 4). (K) The Wright's staining of neutrophils treated with PBS, BRLPs, and CRLPs, respectively. Scale bar, 10 µm. The data were presented as mean ± SEM (*n* = 3) unless otherwise stated. ^*^ *p *< 0.05, ^**^ *p *< 0.01, and ^***^ *p *< 0.001. The abbreviation “ns” indicated no significant difference.

Liposomes (LPs) and cyclic peptide moiety c(RGDfk)‐anchored liposomes (RLPs) were synthesized using the thin film deposition method of lipid. Transmission Electron Microscopy (TEM) demonstrated that both LPs and RLPs had a uniform spherical shape with a smooth surface (**Figure** **1**A,B). The diameters of LPs and RLPs measured by dynamic light scattering (DLS) were 118.2 ± 0.2  and 118.4 ± 1.6 nm, respectively (Figure 1C). Additionally, the zeta potential of RLPs (−22.8 ± 0.4 mV) was significantly higher than that of LPs (−36.4 ± 1.7 mV) (Figure 1D), likely due to the positive charge of c(RGDfk) under physiological conditions.^[^ ^9^ ^]^ Favorable stability of RLPs in water was found during a span of 7 days, with diameter and zeta potential maintaining ≈100 nm and −22 mV, respectively (Figure 1E).

For evaluating the targeting ability of RLPs, neutrophils isolated from mouse bone marrow at the density of 10^6^/mL were incubated with lipophilic dye‐labeled nanoparticles for 1 h at 37 °C. Flow cytometry study revealed that the mean fluorescence intensity (MFI) of neutrophils in the RLPs‐treated group was threefold greater than that of the LPs‐treated group (Figures 1F and S1A,B, Supporting Information). In addition, the confocal laser scanning microscope (CLSM) images further demonstrated the fluorescence intensity of RLPs (green) was stronger than that in the LPs‐treated group (Figure 1G), indicating a superior active targeting capability of RLPs toward neutrophils.^[^ ^10^ ^]^

Two drug candidates, namely the BMS‐202 and CFZ, were carefully chosen and loaded into RLPs, respectively. The BMS‐202 loaded RLPs (BRLPs) and CFZ‐loaded RLPs (CRLPs) have comparable sizes, but the zeta potential of BRLPs was significantly higher than that of CRLPs (Figure 1H,I). The encapsulation efficiencies of CFZ and BMS‐202 in RLPs were 64.0% ± 0.9% and 73.2% ± 0.4%, respectively. The release of carfilzomib and BMS‐202 from CRLPs and BRLPs was conducted in PBS containing 0.5% Tween 80 at pH 7.4, respectively. The results showed that both BRLPs and CRLPs exhibited slow‐release characteristics (Figure S2, Supporting Information). The results of the CCK8 assay demonstrated that both CRLPs and BRLPs had negligible cytotoxicity to neutrophils isolated from mouse bone marrow with a drug concentration ranging from 15 to 120 nmol L^−1^ (Figure 1J). The viability of neutrophils exposed to 120 nmol L^−1^ of drugs encapsulated in CRLPs and BRLPs exhibited a slight decrease over time, with viability values declining to ≈80% after 8 h of incubation (Figure S3, Supporting Information). Furthermore, neither BRLPs nor CRLPs impaired the chemotactic migratory capacity of neutrophils in response to CXCL12 following senescence (Figure S4, Supporting Information). No remarkable morphological changes of neutrophils were found in either CRLPs or BRLPs treated group compared with the blank group by Wright's staining (Figure 1K).

### 2.2 The Neutrophils Hijacking Ability of RLPs In Vitro and In Vivo

**Figure 2:**
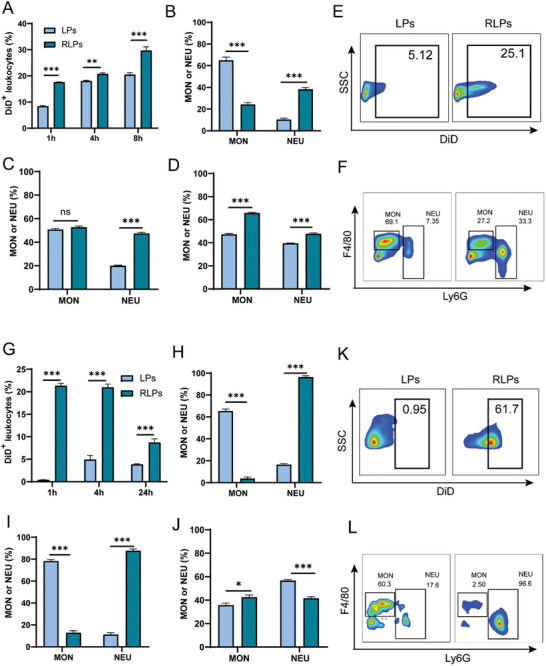
In vitro and in vivo hijacking effects of RLPs on neutrophils. Neutrophil hijacking of RLPs in vitro (A–F) and in vivo (G–L). (A) The proportions of DiD^+^ cells in leukocytes after DiD‐RLPs were incubated with the whole blood for various time points in vitro. (B–D) The proportions of neutrophils (NEU) and monocytes (MON) in DiD^+^ leukocytes after DiD‐RLPs were incubated with the whole blood for 1 h (B), 4 h (C) and 8 h (D), respectively. Representative flow cytometry plots of DiD^+^ leukocytes (E), neutrophils and monocytes (F) after DiD‐RLPs were incubated with the whole blood for 1 h. (G) The percentages of DiD^+^ cells in leukocytes in the peripheral blood at different time points after DiD‐RLPs injection in vivo. The percentages of neutrophils or monocytes in DiD^+^ leukocytes at 1 h (H), 4 h (I) and 8 h (J) after intravenous administration of RLPs. Representative flow cytometry plots of DiD^+^ leukocytes (K), neutrophils and monocytes (L) at 1 h after intravenous administration of RLPs. The LPs‐treated group was included as a control. The data were presented as mean ± SEM (*n* = 4). ^*^ *p *< 0.05, ^**^ *p *< 0.01, and ^***^ *p *< 0.001. The abbreviation “ns” indicated no significant difference.

The whole blood from mice was incubated with DiD‐labeled LPs or RLPs at 37 °C for various durations. Given the typical lifetime of peripheral neutrophils is 7–9 h, the maximum incubation time selected for in vitro incubation is 8 h.^[^ ^8^, ^11^ ^]^ It was found that a higher percentage of blood cells could be labeled with RLP nanoparticles after incubating for 1, 4, and 8 h compared to the LPs labeled group (**Figure** **2**A,E). To pinpoint the specific population of the dye‐positive cells, anti‐mouse CD45, F4/80, and Ly6G antibodies were used to identify monocytes (CD45^+^Ly6G^−^F4/80^dim^ population) and neutrophils (CD45^+^Ly6G^+^ population) (Figure S5, Supporting Information).^[^ ^12^ ^]^ After incubation for 1 h, ≈38.7% of RLP‐positive cells were neutrophils, while only 25.5% of RLP‐positive cells were monocytes. However, for the liposome without RGD labeling, the monocyte and neutrophil percentages of the LPs‐positive cells were 61.4% and 10%, respectively (Figure 2B,E,F). Neutrophil uptake of RLPs reached rapid saturation within a short period, after which the proportion of RLPs taken up by monocytes increased over time (Figure 2B–D). These findings suggest that the selective binding of RLPs toward neutrophils is owing to active targeting, which laid the foundation for neutrophil hijacking in vivo. In addition, the level of CXCR4, which was the marker of neutrophil senescence, was increasingly expressed on neutrophils' surface with incubation time extended. Interestingly, the CXCR4 expression levels for BRLPs or CRLPs‐treated groups at different time points did not exhibit any significant variation compared with the PBS‐treated group (Figure S6, Supporting Information), indicating the nanoparticle treatment had mild effects on neutrophil senescence.

Inspired by the in vitro neutrophil targeting capability of RLPs, the in vivo neutrophil hijacking was also evaluated. Blood was collected from both LPs and RLPs treated groups at 1 , 4 , and 24 h following intravenous injection, flow cytometry was employed to analyze the proportion of nanoparticle‐positive cells and subpopulations of those cells. Flow cytometry analysis revealed that the proportion of RLPs‐positive leukocytes was consistently higher than the percentage of LPs‐positive cells at all time points (Figure 2G). And nearly all of RLPs positive cells were neutrophils (98.7%) 1 h post‐injection, while 64.5% of the LPs positive cells were monocytes rather than neutrophils, suggesting the excellent neutrophil hijacking effect of RLPs (Figure 2G,K,L). In addition, the percentages of neutrophils among RLPs positive cells decreased from 98.7% to 42.5% at 24 h post‐injection, likely due to neutrophil senescence and their subsequent migration from the bloodstream to other tissues (Figure 2H–J). With regard to the uptake efficiencies of LPs and RLPs in different blood cell types, the results indicated that within 4 h, neutrophils not only comprised the largest proportion of cells binding with RLPs but also took up the highest amount of these particles. In contrast, monocytes exhibited the greatest uptake of LPs at 4 h. However, by the 24 h mark, this trend reversed. This observation was consistent with the typical lifespan and functional behavior of neutrophils, which initially showed high uptake of RLPs in the bloodstream but gradually exited circulation as they aged. Monocytes, on the other hand, might take longer to interact with RLPs and maintain a more sustained uptake over time (Figure S7, Supporting Information).

### 2.3 The Bone Marrow Targeting Ability of RLPs

**Figure 3:**
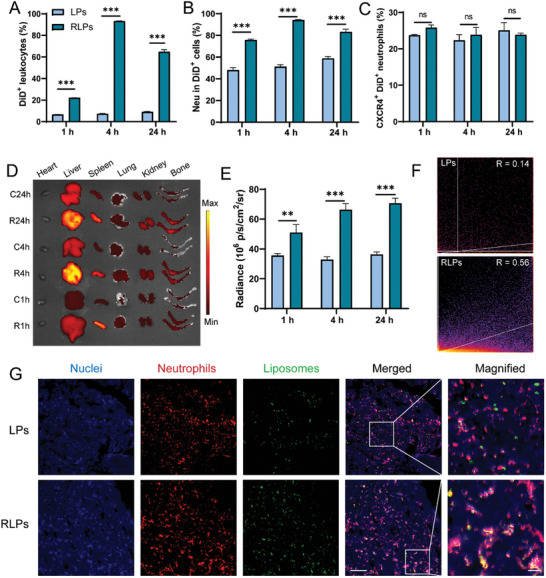
The targeting ability of RLPs to bone marrow. The percentages of DiD^+^ cells in leukocytes (A), percentages of neutrophils in DiD^+^ cells (B), and percentages of CXCR4^+^ cells in DiD^+^ neutrophils (C) in bone marrow at 1 , 4 , and 24 h after intravenous administration of DiD‐ RLPs. (D,E) Representative fluorescence images of key organs (D) and the accompanying quantitative assessment of the fluorescence intensity of femur and tibia (E) at 1 , 4 , and 24 h following intravenous administration of DiD‐LPs or RLPs. The Pearson's R value of colocalization of DiD‐labeled liposomes and neutrophils (F) and representative fluorescence images of bone marrow slices at 4 h after intravenous injection of DiD‐liposomes (G). Blue, DAPI‐stained nuclei. Red, neutrophils stained by PE‐labeled anti‐Gr‐1 antibodies. Green, DiD‐labeled LPs or RLPs. Scale bars were 50 µm in the merged images and 10 µm in the magnified images, respectively. The data were presented as mean ± SEM (*n* = 4). ^*^ *p *< 0.05, ^**^ *p *< 0.01, and ^***^ *p *< 0.001. The abbreviation “ns” indicated no significant difference.

To assess the bone marrow targeting efficacy of RLPs in vivo, DiD‐labeled LPs and RLPs were administered intravenously to mice. Flow cytometry analysis revealed that the proportion of nanoparticle‐positive cells in the RLPs‐treated group was significantly higher than that of the LPs‐treated group at 1 , 4 , and 24 h post‐injection, indicating the effective targeting capability of the c(RGDfk) moiety on the liposome surface (**Figure** **3**A). Among the nanoparticle‐positive cells (DiD^+^), neutrophils represented the largest percentage in both treatment groups (over 70% in the RLPs‐treated group and over 40% in the LPs‐treated group), with a significantly larger proportion in the RLPs‐treated group compared with the LPs‐treated group (Figure 3B). Additionally, the expression of the neutrophil homing molecule CXCR4 was unaffected by either liposome formulation at 1 , 4 , and 24 h (Figure 3C).

The tissue distribution of RLPs and LPs was also evaluated. It was shown that the fluorescence intensity of the femur and tibia in both treatment groups increased over time following injection (Figure 3D,E). The fluorescence intensity of bone marrow in the RLPs‐treated group was roughly twice as high as that of the LPs‐treated group 24 h post‐injection, which further confirmed the successful targeting effect as shown in Figure 3A. Please note that the fluorescence intensity of livers and spleens in RLPs‐treated group was much stronger than that in LPs‐treated group for all the injection time points (Figure S8A–C, Supporting Information), which might be explained by the blood circulation time of RLPs (5.7 ± 0.2 h) was significantly shorter than that of LPs (9.8 ± 0.7 h) (Figure S9A,B, Supporting Information), the accumulation of nanoparticles in the reticuloendothelial system was normally negatively correlates with the circulation time.^[^ ^13^ ^]^ However, despite the more accumulation in the liver and spleen, RLPs still harbored a superior capability for bone marrow targeting as compared with LPs. Myeloma originates in the bone marrow and is typically confined to this area, progressing slowly in most cases. However, it can become highly invasive and metastasize to extramedullary sites, including the liver and spleen, presenting significant clinical challenges and often correlating with a poor prognosis.^[^ ^14^ ^]^ RLPs could potentially target organs besides bone marrow such as the liver and spleen, which might open an alternative strategy for extramedullary myeloma treatment.

Fluorescence imaging of bone marrow cells was performed to determine the precise cell types associated with nanoparticles by injecting DiD‐labeled nanoparticles alongside a neutrophil marker (PE‐labeled anti‐Gr‐1 antibodies) into mice. Results revealed that the RLPs fluorescence signal exhibited a better co‐localization with neutrophils compared to LPs, as evidenced by a higher Pearson's R‐value of 0.56 for RLPs versus 0.14 for LPs (Figure 3F,G). This result matched well with the flow cytometry (Figure 3A) and *ex vivo* imaging (Figure 3D) results, further verifying that RLPs could target bone marrow through a neutrophil hijacking strategy.

What's more, in neutrophil‐depleted mice, RLPs accumulated more in the liver and spleen, with reduced distribution in the bone marrow. The bone marrow RLPs fluorescence intensity in the control group was ≈1.8 times higher than that in the neutrophils‐depleted group, underscoring the key role of neutrophils in directing RLPs to the bone marrow and reducing RLPs uptake by phagocytes in the liver and spleen (Figure S10, Supporting Information).

### 2.4 Pharmacodynamics Against MM

**Figure 4:**
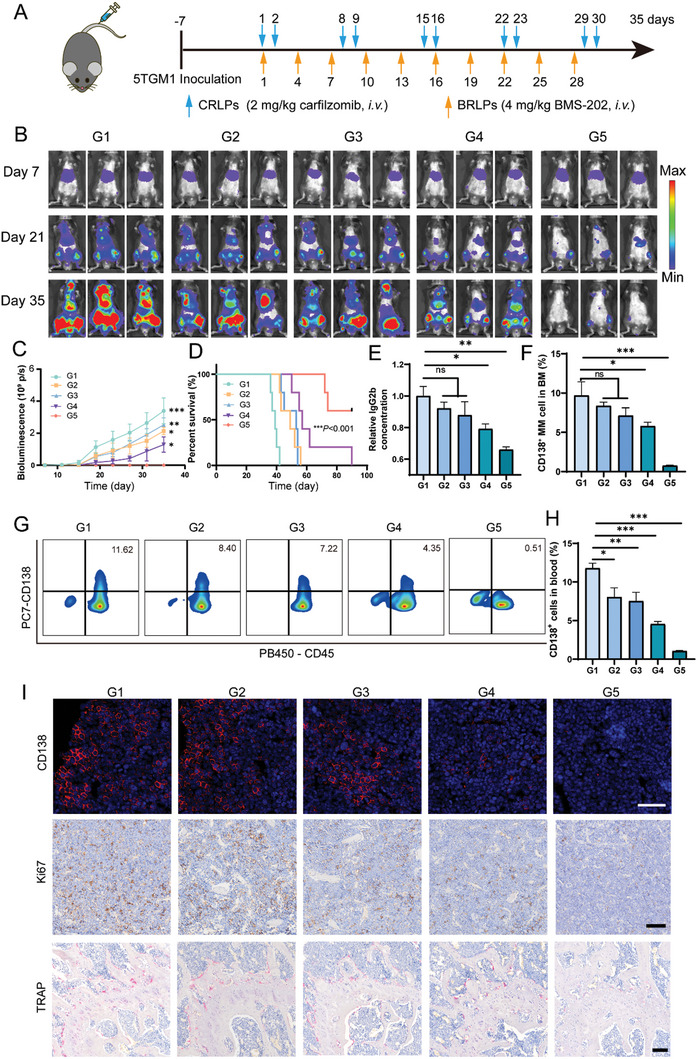
Pharmacodynamics experiment against MM. (A) The schematic timeline for pharmacodynamics experiment. Tumor bearing mice were treated with PBS, BLPs+CLPs, BRLPs, CRLPs, and BRLPs+CRLPs, respectively. (B,C) The representative bioluminescence images and corresponding quantitative analysis of the bioluminescence signal from MM mouse models which received different treatments. (D) The survival curves of mouse models after different treatments. (E) The concentrations of serum IgG2b in different treatment groups, normalized to the levels tested in the BRLPs+CRLPs group at the study endpoint. The percentages of CD138^+^ myeloma cells in the bone marrow (F) and in the peripheral blood (G, H) analyzed by flow cytometry. (I) Representative images of femur tissue slices from different treatment groups after CD138 immunofluorescent staining, ki67 immunohistochemical staining, and TRAP staining. Scale bar, 100 µm. G1, the PBS group. G2, the BLPs+CLPs group. G3, the BRLPs group. G4, the CRLPs group. G5, the BRLPs+CRLPs group. The data were presented as mean ± SEM (*n* = 5). ^*^ *p *< 0.05, ^**^ *p *< 0.01, and ^***^ *p *< 0.001. The abbreviation “ns” indicated no significant difference.

MM‐bearing mice models were established by intravenously inoculating one million 5TGM1‐luc cells for each six‐week‐old C57BL/KaLwRij mouse. The dosing regimen was presented as **Figure** **4**A. The mice were given five cycles of *i.v*. administration of BRLPs+CRLPs during the treatment course. Firstly, the luminescence intensity of 5TGM1‐luc cells representing myeloma burden was quantified as normalized total flux using an in vivo imaging system (IVIS). As compared to the PBS group, all the treatment groups showed some anti‐myeloma efficacy against myeloma. Among them, the combined group of BRLPs and CRLPs had the strongest anti‐myeloma efficacy (Figure 4B,C), which thus prolonged median survival time of MM‐bearing mice to “undefined” (the point of termination was 90 days) (Figure 4D). As myeloma cells could secrete monoclonal immunoglobulin, the serum IgG2b level was used as an alternative indicator of the myeloma burden. Enzyme‐linked immunosorbent assay (ELISA) was utilized to quantify the serum IgG2b level, the results showed that BRLPs + CRLPs treatment led to a significant reduction of serum IgG2b, compared with CRLPs and BRLPs single treatment groups, respectively (Figure 4E). Furthermore, the proportions of 5TGM1 cells in karyocytes could also be regarded as another indicator of myeloma burden, it was found that the percentage of 5TGM1 cells was the lowest in both peripheral blood and bone marrow in BRLPs+CRLPs treatment group as compared with the rest groups (Figure 4F–H). The immunofluorescence staining for CD138 was used to visualize the distribution of myeloma cells in the bone marrow. Consistently, BRLPs+CRLPs treatment led to the weakest fluorescence compared to other treatment groups (Figure 4I). Lastly, ki67 was utilized as a marker for MM cell proliferation monitoring in the bone marrow. The fewest ki67 positive cells were presented in the bone marrow of BRLPs+CRLPs treatment group, indicating the lowest proliferation of myeloma cells (Figure 4I). Bone disease is a prevalent manifestation in myeloma patients, with ≈80% of individuals exhibiting associated bone damage. The underlying mechanism behind bone damage of MM is the imbalance between osteoblasts and osteoclasts, the increased osteoclast activity elicits osteolysis, and interaction between osteoclasts and myeloma cells facilitates the myeloma progression.^[^ ^15^ ^]^ To this end, tartrate‐resistant acid phosphatase (TRAP) staining, which specifically labels the osteoclasts as red color, was used to measure the activity of osteoclasts. Results showed that BRLPs+CRLPs treatment resulted in a significantly lower population of osteoclasts than other control groups (Figure 4I). It was previously reported that CCL3 is associated with the proliferation and migration of myeloma cells, and it mediates osteolytic lesions by activating specific receptors on osteoclast precursors or mature osteoclasts, and its level in vivo is associated with the prognosis of MM patients.^[^ ^16^ ^]^ What's more, the results revealed a significant reduction in CCL3 levels only in the mice treated with the combination of CRLPs and BRLPs, whereas no statistically significant differences were observed between other treatment groups and the PBS group. This suggested that the combined therapy achieved a more remarkable therapeutic effect (Figure S11A, Supporting Information), which correlated with the TRAP staining results of osteoclasts as shown in Figure 4I. Additionally, whole blood was collected from the mice at the pharmacodynamic endpoint, and neutrophil and platelet counts were determined using a cell counter. The results indicated that the cell counts for all groups were within the normal range, with no significant differences among different treatment groups (Figure S11B,C, Supporting Information). To sum up, the combination of BRLPs and CRLPs effectively reduced the myeloma burden, prohibited the progression of myeloma and prolonged the overall survival of MM‐bearing mice models. Additionally, osteoclasts were reported to be highly involved in the immunosuppressive state of the myeloma microenvironment,^[^ ^15^ ^]^ which suggested that the immune microenvironment could be further studied to understand the underlying mechanism driving the therapeutic benefits of the combination treatment with BRLPs+CRLPs.

### 2.5 The Microenvironment Modulating Effect

Macrophages with the M2 phenotype are abundant in the tumor microenvironment of MM patients and play a crucial role in promoting the progression, metastasis, and drug resistance of MM.^[^ ^17^ ^]^ Consequently, therapies designed to convert M2‐type macrophages into more immune‐activated M1‐type macrophages are promising for the management of myeloma. CFZ, an FDA‐approved medication primarily utilized for the treatment of RRMM, has been shown to effectively induce a phenotypic shift in M2 type macrophages in both in vitro and in vivo settings. Additionally, the combination of CFZ with a checkpoint inhibitor showed excellent efficacy for solid lung cancers.^[^ ^18^ ^]^

**Figure 5:**
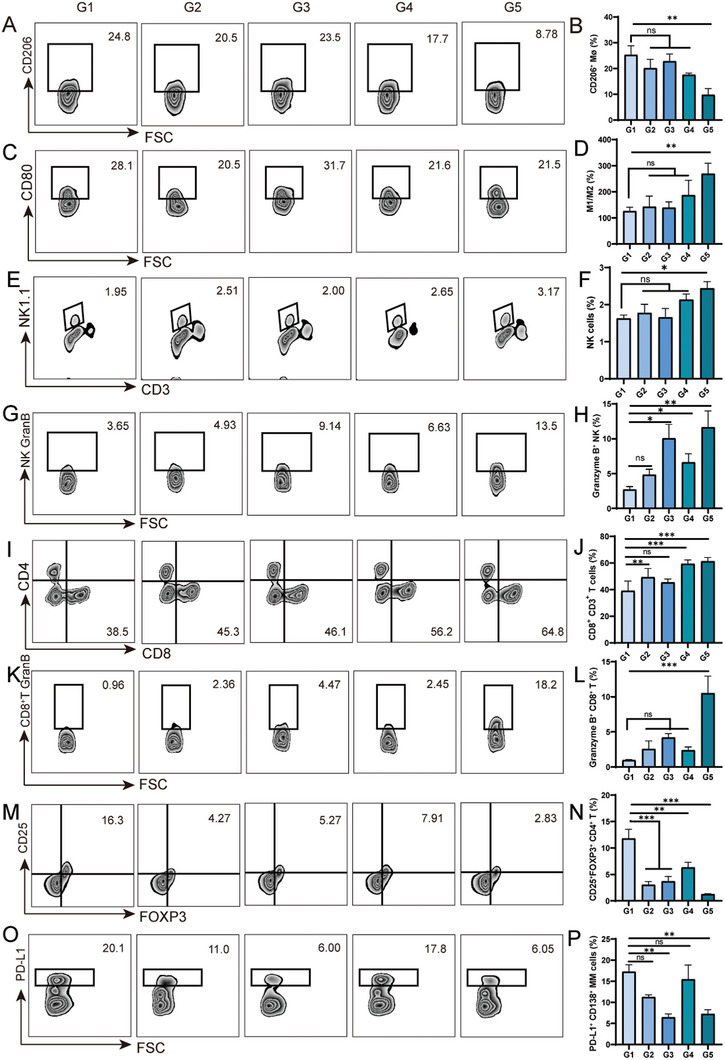
The microenvironment modulating effect. (A,B) The FACS plots and the corresponding quantitative analysis of CD45^+^CD11b^+^F4/80^+^CD206^+^ macrophage cells. (C,D) The FACS plots of CD45^+^CD11b^+^F4/80^+^CD80^+^ macrophages (M1), alongside quantitative analysis of the M1/M2 (CD45^+^CD11b^+^F4/80^+^CD206^+^) cell ratio. (E, F) The FACS plots and quantitative analysis of CD45^+^CD3^−^NK1.1^+^ NK cells. (G,H) The FACS plots and the corresponding quantitative analysis of CD45^+^CD3^−^NK1.1^+^GranB^+^ NK cells. (I,J) The FACS plots and quantitative analysis of CD45^+^CD3^+^CD8^+^ T cells. (K,L) The FACS plots and the corresponding quantitative analysis of CD45^+^CD3^+^CD8^+^GranB^+^ T cells. (M,N) The FACS plots and the corresponding quantitative analysis of CD45^+^CD3^+^CD4^+^CD25^+^FOXP3^+^ Treg cells. (O,P) The FACS plots and the corresponding quantitative analysis of CD45^+^CD138^+^PD‐L1^+^ myeloma cells. G1, the PBS group. G2, the BLPs+CLPs group. G3, the BRLPs group. G4, the CRLPs group. G5, the BRLPs+CRLPs group. The data were reported as mean ± SEM (*n* = 6). ^*^ *p* < 0.05, ^**^ *p* < 0.01, and ^***^ *p* < 0.001. The abbreviation “ns” denoted no significant difference.

The in vitro results illustrated that CFZ alone reversed the IL‐4‐pretreated bone marrow‐derived macrophages (BMDMs) into M1 phenotype, resulting in up‐regulation of M1 marker CD86 and the down‐regulation of M2 marker CD206, as well as increasing the secretion of the pro‐inflammatory cytokines, such as IL‐1*β*, IL‐6, and TNF‐*α* (Figure S12A–E, Supporting Information). Inspired by the efficacy of CFZ on macrophages, the combination of CRLPs and BRLPs was also tested in vivo. After *i.v*. administration of drug‐loaded liposomes, the subtypes of bone marrow cells in MM‐bearing mice were analyzed by flow cytometry. The CRLP treatment resulted in a notable decrease in M2 phenotype macrophages (CD206 positive) in bone marrow, from 25.24% to 17.54%. More excitingly, BRLPs+CRLPs treatment resulted in the lowest frequency (9.76%) of M2 phenotype macrophages (**Figure** **5**A,B). Moreover, the ratio of the M1 phenotype to the M2 phenotype was the highest in the BRLPs+CRLPs group among all the treatment groups (Figure 5C,D). The results demonstrated the synergistic effect of BRLPs and CRLPs in terms of inducing the polarization of pro‐tumorigenic M2 macrophages to tumoricidal M1 phenotype.

In addition to comparing macrophage percentages in the bone marrow, natural killer (NK) cells were also evaluated following various treatments. As shown in Figure 5E,F, BRLPs+CRLPs treatment boosted the NK cells percentage up to 2.44% in the bone marrow. Moreover, the ratio of granzyme B positive NK cells, indicative of cytotoxic phenotypes, exhibited a significant increase in the BRLPs+CRLPs treatment group compared to the single treatment groups and the PBS control group (Figure 5G,H). Those results could be attributed to the fact that CFZ downregulated the expression of human leukocyte antigen class I on myeloma cells, which is correlated with the enhancement of NK cells mediated lysis and NK cell degranulation.^[^ ^19^ ^]^ In addition, the PD‐L1 inhibitors in BRLPs also played an important role in transforming naïve NK cells into tumoricidal phenotype, which might be attributed to the interruption of the PD‐1/PD‐L1 axis between NK cells and MM cells.^[^ ^20^ ^]^

Given macrophages and NK cells held the capacity to re‐educate T cells for anti‐tumor immunity, the levels of immuno‐stimulating cytotoxic T lymphocytes (CTLs) and immuno‐suppressive regulatory T cells (Tregs) were assessed.^[^ ^21^ ^]^ The results presented in Figure 5I,J indicate that the percentage of CD8+ T cells within the T cell population for the BRLPs+CRLPs treatment group reached 61.30%, nearly double that of the control group. Granzyme B secretion is the hallmark of CD8^+^ T cell activation, we thus compared its expression among the treatment groups. It was found that the proportion of Granzyme B positive CD8^+^T cells, namely the CTLs, was significantly higher than the rest groups (Figure 5K,L), indicating that BRLPs+CRLPs treatment significantly boosted the tumoricidal activity of CD8^+^T cells. On the contrary, the percentage of immune‐suppressive FOXP3‐expressing Tregs was dramatically decreased compared with other groups, which is opposite to the trend of cytotoxic T cells change, suggesting the dual effect of BRLPs+CRLPs (Figure 5M,N). The cytokine levels correlated with the anti‐tumor effect in bone marrow tissues were explored using in situ immunofluorescence staining. Consistent with the changes observed in immune cells, the fluorescence intensity of IL‐1*β*, IL‐6, and TNF‐*α* in bone marrow tissues, along with their concentrations in mouse serum as measured by ELISA, were highest in the BRLPs+CRLPs group (Figures S13 and S14, Supporting Information). This further supports the immune‐stimulating effect of the combination treatment.

Lastly, PD‐L1 receptors on the surface of myeloma cells were also evaluated. Results in Figure 5O,P showed that BRLPs+CRLPs treatment significantly decreased the percentage of PD‐L1 expression down to 7.27%, compared to 17.25% in the PBS group, which was mainly owing to the inhibitive effect of BMS‐202 on the expression of PD‐L1 of myeloma cells.^[^ ^22^ ^]^ High expressions of PD‐L1 on tumor cells facilitate the exhaustion of cytotoxic CD8^+^ T cells,^[^ ^23^ ^]^ so the down‐regulation of PD‐L1 expression partially explains the fact that the cytotoxic CD8^+^ T cell percentage was enhanced in BRLPs+CRLPs treatment group.

To sum up, the mechanism study revealed that the two major active pharmaceutical ingredients in BRLPs and CRLPs played important roles in synergistically transforming the phenotype of macrophages from immune‐suppressive M2 type to immune‐promoting M1 type, enhancing the activity of NK cells, down‐regulating the PD‐L1 expression on myeloma cells, decreasing the proportion of Tregs, and increasing the proportion and activity of CTLs, which collectively explained the excellent in vivo anti‐tumor efficacy.

### 2.6 Safety Evaluation

**Figure 6:**
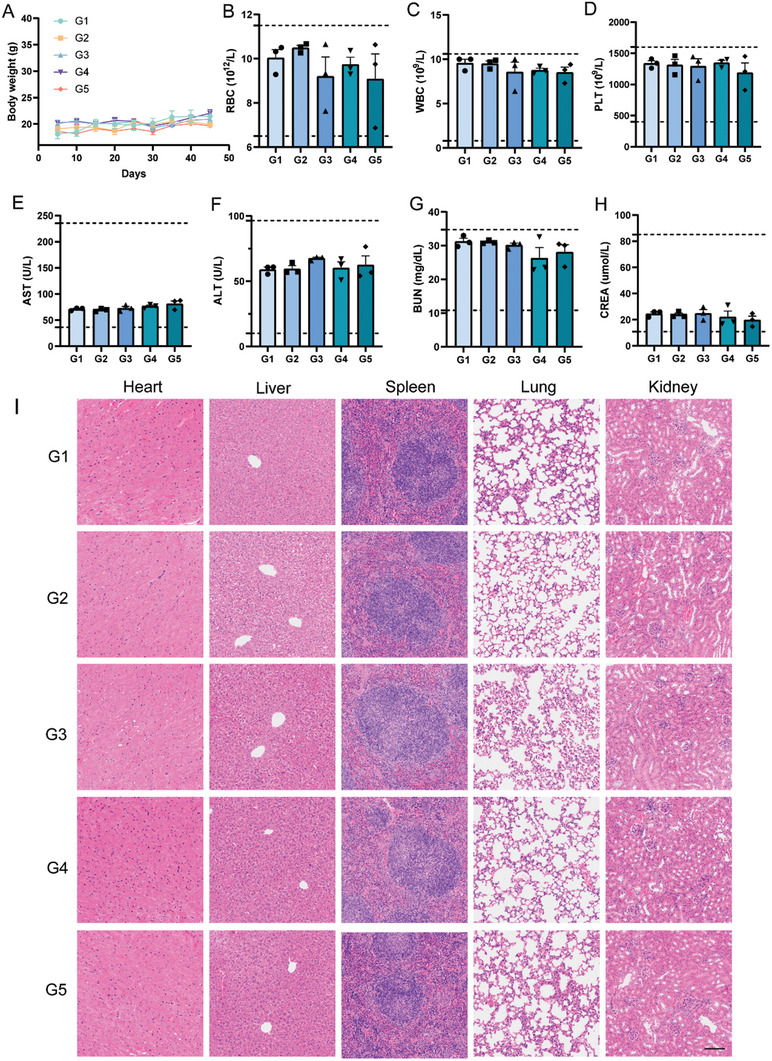
Biocompatibility evaluation. (A) The body weight changes over time after various treatments. The blood cell counting after receiving different treatments, including RBC (B), WBC (C) and PLT (D). (E‐H) The blood chemistry panel, includes AST (E), ALT (F), BUN (G), and CREA (H). (I) Representative H&E staining image of major organ slices for histological analysis. Scale bar, 100 µm. G1, the PBS group. G2, the BLPs+CLPs group. G3, the BRLPs group. G4, the CRLPs group. G5, the BRLPs+CRLPs group. The data were presented as mean ± SEM (*n* = 3).

In order to verify the biosafety of BRLPs+CRLPs, the body weight, major blood cell counting, and biochemical test of normal mice receiving BRLPs plus CRLPs treatment were conducted. The body weight of the mice showed a slight increase across all treatment groups (**Figure** **6**A). Hematology analysis showed RBC, WBC, and PLT counts were all in the normal range (Figure 6B–D). All the treatment groups did not show any notable variations in terms of the levels of AST, ALT, BUN, and CREA (Figure 6E–H). The levels of myocardial enzymes such as CK, CK‐MB, LDH, and LDH‐1 in all treatment groups were also within the normal range, suggesting that the drugs administered to the mice did not induce significant cardiotoxicity (Figure S15, Supporting Information).^[^ ^24^ ^]^ Finally, the H&E staining of key organs showed that the mice that received the BRLPs+CRLPs treatment had no obvious morphology change as compared with the PBS treatment group (Figure 6I). In summary, the results demonstrated a satisfactory safety profile of the combination treatment with BRLPs and CRLPs.

As related clinical trials have been suspected due to the low remission rate found in RRMM patients when treated with a single PD‐1 inhibitor or the safety concern when combined with the immunomodulator lenalidomide,^[^ ^4^, ^5^ ^]^ researchers are still making efforts to explore an alternative approach to enhance the efficacy and safety of PD‐1/PD‐L1 inhibitors for RRMM patients. Thus, the combination treatment with BMS‐202 and CFZ proposed in this study offered an effective and safe approach to enhance the efficacy of PD‐1/PD‐L1 blockade for myeloma treatment, which possesses promising clinical translational potential.

## 3 Conclusion

Here we developed a novel c(RGDfk)‐functionalized liposome formulation that could deliver drug combination to bone marrow via neutrophil hijacking, which resulted in the modulation of the local myeloma microenvironment toward a more favorable direction for therapy. As one of the most important drugs for RRMM, CFZ not only directly kills MM cells, but also modulates the tumor microenvironment to increase NK cell‐mediated MM cell lysis. BMS‐202 is a small‐molecule PD‐L1 inhibitor that disrupts the PD‐L1 dimerization, which exhibited several advantages over the traditional antibody‐based checkpoint inhibitor, such as higher stability, improved tumor penetration, lower immunogenicity, and facile production process. Therefore, for the first time, the combination of CFZ with BMS‐202 is extremely meaningful in terms of modulating the tumor microenvironment toward more favorable directions for MM treatment. Both in vitro and in vivo studies showed that RLPs could recognize neutrophils in the whole blood and be delivered to bone marrow via neutrophil hijacking. The In vivo therapeutic efficacy against MM was performed to show BRLPs plus CRLPs treated group significantly modulated myeloma microenvironment, inhibited myeloma growth, and prolonged the lifespan of murine models. This study introduced an alternative drug combination for myeloma immunotherapy and demonstrated the efficacy of a neutrophil hijacking strategy in overcoming significant challenges in MM treatment. These findings also pave the way for new avenues in drug delivery with high efficiency to the bone marrow.

## 4 Experimental Section

### Materials, Cells and Mice

The hydrogenated soy phosphatidylcholine (HSPC), Cholesterol, 1,2‐distearoyl‐sn‐glycerol‐3‐phosphoethanolamine‐N‐succinimidyl (polyethylene glycol)‐2000 (DSPE‐PEG2000‐NHS) and 1,2‐distearoyl‐sn‐glycero‐3‐phosphoethanolamine‐N‐ [methoxy (polyethylene glycol)‐2000] (DSPE‐mPEG2000) were purchased from AVT Pharmaceutical Tech Co., Ltd. (Shanghai, China). The c(RGDfk) was obtained from GL Biochem (Shanghai) Ltd. The DiD dye (C1039) and cell membrane red fluorescence staining kit (DiI) were bought from Beyotime Biotechnology (Shanghai, China). CXCL12 cytokines, BMS‐202, and Carfilzomib (CFZ) were purchased from MedchemExpress (Monmouth Junction, NJ, USA). Recombinant murine macrophage colony‐stimulating factor (315‐02) was bought from PeproTech (Cranbury, NJ, USA). All enzyme‐linked immunosorbent assay (ELISA) kits (including IL‐6, IL‐1*β*, TNF‐*α*, CCL3, and IgG2b) were purchased from Shanghai Jianglai Biotechnology Co., Ltd (Shanghai, China). The DAPI and CCK8 kit were obtained from keyGEN Biotech Co., Ltd. (Jiangsu, China). The Wright's staining kit and TRAP staining kit were bought from Beijing Solarbio Science & Technology Co., Ltd. (Beijing, China). The antibodies for flow cytometry staining and neutrophils depletion were provided by Biolegend (California, the USA).

Six‐week‐old C57BL/6J male mice were ordered from Shanghai SLAC Laboratory Animal Co., Ltd. And C57BL/KaLwRij mice were kindly provided by Professor Zhiqiang Liu from Shandong Cancer Hospital and Institute. They were housed in a sterile animal house at the School of Pharmacy, Fudan University with food and water ad libitum. The animal experiment protocol was approved by the Ethics Committee of Fudan University (2022‐03‐YJ‐PZQ‐08) and also complied with the requirements of the International Code of Animal Ethics.

The 5TGM1‐Luciferase (5TGM1‐luc) cell line was donated by Professor Yuhuan Zheng of Sichuan University and maintained in RPMI 1640 medium (Corning, 10040CV) with 10% FBS (CellCo, FBS003) and 0.01mg mL^−1^ blasticidin (Gibco, A1113903), under a humidified environment of 5% CO2 and 37 °C.

### Preparation and Characterizations of RLPs

DSPE‐PEG2000‐NHS and c(RGDfk) were dissolved in 3 mL of Dimethylformamide (DMF, Sinopharm Chemical Reagent Co., Ltd.) at a molar ratio of 1:1.5 with the pH set to 8.0 by addition of N, N‐Diisopropylethylamine (DIPEA, Sinopharm Chemical Reagent Co., Ltd.), and the concoction was agitated at ambient temperature for a duration of 24 h. The samples were further dialyzed against distilled water for 48 h using 2000 molecular weight cut‐off size dialysis bags, followed by lyophilization to get the powder form of DSPE‐PEG2000‐c(RGDfk).

Liposomes were formulated utilizing a conventional thin‐film hydration method followed by ultrasonication.^[^ ^25^ ^]^ Specifically, HSPC, cholesterol, and DSPE‐mPEG2000 or DSPE‐mPEG2000‐c(RGDfk) (9/3/1, w/w) with a total mass of 7.5 mg were dissolved in 9 mL of chloroform in a 50 mL round‐bottom flask. The chloroform was removed using vacuum evaporation at 40 °C to form a thin film, followed by hydration with 2 mL of distilled water. Then the resulting suspension was subjected to ultrasonication using a water bath sonicator (120 W) for 30 times to form blank liposomes (LPs) or c(RGDfk) modified liposomes (RLPs). To prepare DiD‐labeled or drug‐loaded LPs or RLPs, the same procedures were performed as preparing blank LPs or RLPs except that DiD or drugs such as CFZ or BMS‐202 were added into the lipid stock solution in chloroform in advance. DiD was added to the lipid mixture in chloroform with a molar ratio at 1:50 (fluorescent dye: HSPC), and BMS‐202 or CFZ was dissolved in a lipids mixture in chloroform with a molar ratio of 11.93% or 2%, respectively. The encapsulation efficiency of the drug was measured by high‐performance liquid chromatography (HPLC, 1200LC, Agilent, California, USA), the detection wavelength was 210 nm for CFZ and 230 nm for BMS‐202, respectively. To evaluate the drug release properties of CRLPs and BRLPs, each formulation was subjected to ultrafiltration at 5000×g for 50 min to remove free drugs. Subsequently, 5 mg of CRLPs and BRLPs were placed in separate dialysis bags with a 10 kDa molecular weight cutoff (MWCO) and immersed in 50 mL of the release medium, which was at least three times the volume needed for a saturated drug solution to ensure sink conditions. The dialysis bags were incubated on a shaking platform at 37 °C. At various time points, 200 µL of the release medium was withdrawn and analyzed by liquid chromatography to determine drug concentrations. The cumulative drug release rates from BRLPs and CRLPs were then calculated.

Cryogenic electron microscopy (cryo‐EM, Tecnai G2 F20; FEI, Eindhoven, Netherlands) was used to characterize the morphology of liposomes. The samples were spotted on carbon‐coated copper grids, which underwent liquid ethane plunging and liquid nitrogen loading. The imaging was conducted at an accelerating voltage of 120 kV.

The diameters and zeta potentials of liposomes were characterized using Malvern ZS90 (ZEN3600 Zetasizer, Malvern, UK). The stability of liposomes in deionized water was investigated by monitoring the particle size and zeta potential every day for 7 days.

### Isolation of Murine Neutrophils

The neutrophils were extracted using the method described in the literature.^[^ ^26^, ^27^ ^]^ Following the euthanasia of normal mice, the femurs were collected from euthanized mice and the bone marrow cavity of the femurs was flushed with FACS solution (PBS buffer supplemented with 2 mmol L^−1^ EDTA and 0.5% BSA) using a 25G needle until they turned whitish. The effluent was collected, passed through a 40 µm cell strainer, and erythrocytes were lysed on ice for 10 min. Afterward, the cell precipitate was collected by centrifugation at 400×g for 5 min and washed with the above buffer three times. Finally, the bone marrow‐derived neutrophils were isolated and purified using a commercialized neutrophil isolation kit (Miltenyi Biotec, 130 097 658) following the manufacturer's protocol.

### The Neutrophils Hijacking Effect of RLPs

Firstly, to test if the liposome formulation with or without targeting moieties can specifically bind to neutrophils in vitro, the extracted neutrophils were incubated with 0.5 mg mL^−1^ of DiD labeled‐LPs or RLPs for 1 h in RPMI 1640 complete medium. Afterward, flow cytometry was used to quantify the particle positive cells. Meanwhile, cell membranes were stained with DiI at a concentration of 2.5 µm for 20 min at 37 °C after incubating with liposomes at various times. Lastly, the cells were fixed using 4% formaldehyde in PBS and stained with DAPI (Biosharp, BL105A) for 5 min. The cells were photographed using a confocal laser scanning microscope (CLSM, Carl Zeiss, LSM710, Oberkohen, Germany). In addition, the mouse peripheral blood cells were incubated with 0.5 mg mL^−1^ of DiD‐RLPs or drug‐loaded RLPs for 1, 4, and 8 h, respectively.^[^ ^28^ ^]^ Afterward, erythrocytes in the whole blood were lysed with ACK buffer (Solarbio, R1010) on ice for 15 min followed by dilution with FACS solution. Cells subjected to live‐dead staining in the single‐stain tube were heated in a 70 °C water bath for 5 min before staining, enabling the differentiation between positive and negative groups. Dead cells were excluded by staining with zombie dye (Biolegend, 423 101) at room temperature for 15 min, followed by blocking of Fc segment receptor with anti‐CD16/32 antibody on ice for 30 min. Subsequently, 0.25 µL of FITC anti‐mouse CD45 (0.5 mg mL^−1^), 1.25 µL of BV650 anti‐mouse F4/80 (0.2 mg mL^−1^), 0.6 µL of PE anti‐mouse LY6G (0.2 mg mL^−1^), and 0.6 µL of BV605 anti‐mouse CXCR4 (0.2 mg mL^−1^) antibodies were added to the above cells and incubated on ice for 30 min. After staining, the cells were washed with PBS. Flow cytometry was used to analyze the cells and data processed by Flowjo software (BD, the USA).

To evaluate the hijacking ability of RLPs toward neutrophils in vivo, 100 µL of DiD‐LPs or RLPs at the concentration of 4 mg mL^−1^ were administered intravenously into normal health mice through *i.v*. injection. Peripheral blood cells were collected by postorbital puncture at 1 , 4 , and 24 h post‐administration, respectively. The erythrocyte lysis, staining, and data analysis were carried out using the same protocol described above. The uptake efficiency of DiD‐LPs or DiD‐RLPs by different leukocyte populations was determined by multiplying the mean fluorescence intensity (MFI) of DiD‐labeled liposomes in every kind of leukocytes by the corresponding percentage of each leukocyte subset in the blood and normalizing it to the MFI of total leukocytes at each time point.^[^ ^29^ ^]^

### The In Vitro Safety Evaluation of Drug‐Loaded RLPs

For the CCK8 experiment, neutrophils that were extracted from bone marrow as described above were distributed into 96‐well plates at a density of 2×10^5^ cells per well. Subsequently, BMS‐202‐loaded RLPs (BRLPs) or CFZ‐loaded RLPs (CRLPs) containing 15, 30, 60, and 120 nmol L^−1^ BMS‐202 or CFZ were introduced into the well plates and incubated with neutrophils at 37 °C for 1 h. Additionally, we assessed the impact of varying incubation time points on neutrophil cytotoxicity. The BRLPs and CRLPs, each containing 120 nmol L^−1^ of the drug, were introduced into wells and incubated with neutrophils in vitro at 37 °C for 1, 4, and 8 h, respectively. Afterward, 10% CCK8 solution was added to the wells and left to incubate for another 2 h. The absorbance at the wavelength of 450 nm was used for analyzing the cytotoxicity results using a multimode microplate reader (Tecan, Mechelen, Belgium). Neutrophils without any treatment were used as a negative control.

To assess whether BRLPs or CRLPs impact the migratory chemotactic capacity of neutrophils in response to CXCL12 after senescence, neutrophils were isolated from murine bone marrow and incubated with either BRLPs or CRLPs at a concentration of 120 nmol L^−1^ for 1 h. Following incubation, the cells were washed twice with PBS to remove any residual nanoparticles. Next, 5×10^5^ neutrophils were resuspended in 200 µL of culture medium containing 2% FBS and placed in the upper chamber of a 3 µm polycarbonate transwell insert (Corning, 29442‐110). The lower chamber was filled with 500 µL of culture medium containing 250 ng mL^−1^ of CXCL12. The cells were incubated for 4 h in a cell culture incubator, after which the neutrophils that had migrated to the lower chamber were collected and quantified by flow cytometry to determine the CXCL12‐driven migration rate.

For Wright's staining, neutrophils incubated with BRLPs or CRLPs for 1 h were smeared on the slides, followed by fixation with 70% ethanol for 10 min. The slides were then treated with Wright's solution for 3 min, mixed with an equal amount of PBS, and allowed to rest for another 5 min. After several washes in a beaker, the specimens were air‐dried and subsequently observed using an inverted microscope (Olympus, CKX53, Tokyo, Japan).

### The Bone Marrow Targeting Ability of RLPs

The 100 µL of DiD‐labeled LPs or RLPs at the concentration of 4 mg mL^−1^ were injected into the C57BL/6J mice through tail vein injection. The mice were euthanized at different time points, the femoral bone marrow cells were collected and incubated with fluorescent‐labeled antibodies, including FITC anti‐mouse CD45, BV650 anti‐mouse F4/80, PE anti‐mouse LY6G, and BV605 anti‐mouse CXCR4 antibodies using the same protocol described above. Subsequently, the percentages of DiD‐positive neutrophils and the expression levels of CXCR4 in these neutrophils in the bone marrow were assessed.

For visualizing the co‐localization of neutrophils and RLPs in bone marrow, DiD‐RLPs at the DiD dose of 0.02 µg g^−1^ body weight and PE anti‐mouse Gr‐1 antibody at a dose of 0.05 µg g^−1^ body weight were mixed and injected into mice via the tail vein.^[^ ^30^ ^]^ Heart perfusion using 30 mL of PBS followed by 10 mL of paraformaldehyde was conducted 4 h after treatment. Femur samples were harvested and sliced into sections at the thickness of 10 µm, stained with DAPI, and observed under CLSM.

To further investigate the essential function of neutrophils in delivering drugs to the bone marrow, C57BL/6J mice received intraperitoneal injections of anti‐Ly6G antibody, with the dose of antibodies starting from 400 µg, followed by 100 µg every other day for a total of four doses, while a control group received IgG antibody.[^31^] Peripheral blood samples were then collected, and flow cytometry was used to assess the proportion of neutrophils indicated as CD11b^+^Gr‐1^+^ to confirm successful neutrophil depletion in anti‐Ly6G antibody‐treated mice. Following this, 0.4 mg of DiD‐RLPs was injected via the tail vein into both groups. After 4 h, mice were anesthetized, underwent cardiac perfusion, and important organs including hearts, livers, spleens, lungs, kidneys, and femurs were harvested. Fluorescence intensity of DiD was measured using the IVIS system.

### The Effects of CFZ on Macrophage Polarization

The bone marrow‐derived macrophages (BMDM) were isolated using the protocol described previously.^[^ ^32^, ^33^ ^]^ Briefly, bone marrow cells were harvested from the femurs, and ACK buffer was used to lyse the RBCs using the protocol described above. The remaining cells were counted and seeded into six‐well plates at the density of 2 × 10^6^/mL and cultured in DMEM medium supplemented with 20 ng mL^−1^ macrophage colony‐stimulating factor. On day 5, 20 ng mL^−1^ of IL‐4 was added to the culture and incubated for 24 h to induce the formation of M2‐type macrophages. Afterward, 500 nm CFZ was added to stimulate the cells for another 12 h.^[^ ^18^ ^]^ Finally, the cell supernatant was collected, and ELISA was used to quantify the expression of IL‐1*β*, IL‐6, and TNF‐*α*. The cells adhered to the bottom of dishes were collected using a cell scraper. The expression levels of CD86 and CD206 were analyzed using flow cytometry. PE anti‐mouse CD86 antibody staining was performed using the method described above. Subsequently, intracellular BV650 anti‐mouse CD206 antibody staining was carried out using the Cyto‐Fast™ Fix/Perm Buffer Set (Biolegend, 426 803) following the manufacturer's instructions.

### The Pharmacodynamic Experiment

MM‐bearing mice models were established by intravenously inoculating one million 5TGM1‐luc cells for each six‐week‐old C57BL/KaLwRij mouse. After MM‐bearing mice were developed, these mice were randomly divided into 5 groups, namely the PBS group, the BLPs+CLPs group, the BRLPs group, the CRLPs group, and the BRLPs+CRLPs group. For the CLPs/CRLPs group and the combined group, the formulations were administered intravenously at an equivalent CFZ dose of 2 mg per kg of body weight over the initial two days of each week for 5 weeks, while for BLPs/BRLPs group and the combined group, the formulation was administered at an equivalent BMS‐202 dose of 4 mg per kg of body weight every 3 days for 4 weeks.^[^ ^34^, ^35^, ^36^ ^]^ The dosage was slightly reduced in the combined administration of CFZ and BMS‐202 compared to the dosage used alone in the references. The objective was to evaluate whether the targeted delivery system could sustain therapeutic efficacy at lower doses while minimizing potential drug toxicity. Bioluminescent imaging was conducted under the In Vivo Imaging System (IVIS, PerkinElmer) to monitor the progression of MM. The mice in each group were shaved and then intraperitoneally injected with luciferin at the dose of 50 mg kg^−1^ and waited for 10 min before imaging, the luminescence was quantified using LivingImage software. On day 35, peripheral blood samples were collected via post‐orbital puncture, which was used for IgG2b quantification by ELISA. After the therapeutic regimen, 100 µL of PBS was used to lavage the femoral bone marrow, and the collected fluids were analyzed for the concentration of CCL3 using ELISA. In addition, blood and bone marrow cells were also collected and stained with Percp‐cy5.5 anti‐mouse CD45 and PE/Cy7 anti‐mouse CD138 antibodies to quantify the percentage of myeloma cells using flow cytometry.

For the staining of femur sections, the femurs were initially fixed, decalcified, embedded in OCT, and sectioned into 10 µm thick slices. Subsequently, the sections underwent dewaxing and antigen retrieval and then were blocked using a 5% BSA solution. Mouse anti‐CD138 antibodies (Biotechne, AF3190) were applied and incubated overnight at 4 °C, followed by three times washing with PBS buffer and incubated with PE‐linked secondary antibodies (Invitrogen, A‐11079) at 37 °C for 1 h, the slides were then washed again and counterstained with DAPI and examined under an inverted fluorescent microscope (Olympus, CKX53, Tokyo, Japan). For ki‐67 immunohistochemical staining, DAB (Thermo Scientific, 34 002) was added for color development after incubating with the secondary antibody for 10 min, followed by re‐staining with hematoxylin dye (Beyotime, C0107) for 5 min. Osteoclasts were identified by TRAP staining using a TRAP staining kit.

### The Myeloma Microenvironment Modulating Effect of CRLPs Plus BRLPs

The therapeutic regimen was administered as described above. On day 21, blood was collected from the mice via retro‐orbital puncture into 1.5 mL EP tubes, left at room temperature for 30 min, and then centrifuged at 1000 × g for 10 min. The supernatant was subsequently collected for ELISA analysis to measure the levels of IL‐6, IL‐1β, and TNF‐α. Then the mice in all groups were euthanized, and bone marrow cells were extracted from the femurs. Briefly, the samples were filtered through a 40 µm nylon mesh, treated with ACK buffer for RBC lysis, stained with zombie Aqua dye for viability assessment, and blocked with anti‐CD16/32 antibody to prevent nonspecific binding. Subsequently, the cells were resuspended in cell staining buffer and incubated with specific fluorescent‐conjugated anti‐mouse antibodies on ice for 30 min. For macrophages, the staining panel includes Percp‐cy5.5 anti‐mouse CD45, Pacific blue anti‐mouse‐CD11b, AF488 anti‐mouse F4/80, BV650 anti‐mouse CD206, and PE anti‐mouse CD80 antibodies. For regulatory T cells (Tregs), the staining panel includes Percp‐cy5.5 anti‐mouse CD45, FITC anti‐mouse CD3, BV605 anti‐mouse CD4, PE anti‐mouse CD25, and AF647 anti‐mouse FOXP3 antibodies. For cytotoxic T cells, the staining panel includes APC anti‐mouse CD45, FITC anti‐mouse CD3, Percp‐cy5.5 anti‐mouse CD4, BV605 anti‐mouse CD8a, and PE‐Cy7 anti‐mouse GranB antibodies. For natural killer cells, the staining panel includes APC anti‐mouse CD45, FITC anti‐mouse CD3, BV421 anti‐mouse NK1.1, and PE‐Cy7 anti‐mouse GranB antibodies. For myeloma cells, the staining panel includes Percp‐cy5.5 anti‐mouse CD45, PE/Cy7 anti‐mouse CD138, and APC anti‐mouse PD‐L1 antibodies.

For the immunofluorescence staining of bone marrow tissues, the bone marrow slices were prepared using the method mentioned above. Following blocking with 3% BSA buffer, the slices were incubated overnight with primary antibodies, including anti‐IL‐6 antibody (Invitrogen, P620), anti‐IL‐1*β* antibody (Invitrogen, P420B), and anti‐TNF‐*α* antibodies (Invitrogen, PA5‐19810), respectively. Subsequently, corresponding fluorescent dye‐labeled secondary antibodies were applied. After being counterstained with DAPI, the slices were observed using CLSM.

### Pharmacokinetic and Distribution of RLPs In Vivo

1 mg of DiD‐labeled RLPs or LPs were injected into mice through tail vein, 30 µL of blood samples were collected at various time points (5, 15 , 30 min, 1 , 4 , 8 , 12 , and 24 h) through retro‐orbital puncture. Each blood sample was mixed with 70 µL of PBS and added to a black 96‐well plate for fluorescence measurement at excitation/emission wavelengths of 640/670 nm. The fluorescence intensity signal was plotted against time, which was used to calculate the circulating half‐life using DAS 2.0 software.

To investigate the biodistribution of RLPs or LPs in vivo, 1 mg of DiD‐labeled RLPs or LPs was injected intravenously into the mice. At each time point (1, 4 , and 24 h) post‐injection, the mice were anesthetized and underwent cardiac perfusion using 30 mL of PBS, then the major organs including hearts, livers, spleens, lungs, kidneys, and bones were harvested, which were imaged using the IVIS system at Ex/Em = 640 /670 nm.

### The Biocompatibility of BRLPs and CRLPs

Normal mice received different treatments as the pharmacodynamics experiment to test the biocompatibility of BRLPs and CRLPs. The body weights of mice in each treatment group were monitored during the treatment. On day 45, blood samples were obtained from those mice for routine hematological analyses, including red blood cell count, white blood cell count, and platelet count. In addition, blood samples were also collected for hepatorenal function tests with indicators including AST, ALT, BUN, and CREA, as well as myocardial enzymes such as CK, CK‐MB, LDH, and LDH‐1. After that, these mice were euthanized, and key organs including hearts, livers, spleens, lungs, and kidneys were collected for histological examination. The organs were fixed in paraformaldehyde and processed into paraffin sections for hematoxylin and eosin (H&E) staining to evaluate the tissue morphology.

### Statistical Analysis

The data were presented as mean ± standard error of the mean (SEM) and analyzed using GraphPad prism 8. The comparisons between the two groups were calculated using a two‐tailed unpaired Student's t‐test. Differences among multiple groups were achieved using one‐way ANOVA with Tukey's correction for post hoc comparisons. The Kaplan Meier curves were generated and the Log‐rank (Mantel‐Cox) test was performed to statistically assess the survival rates. The *p*‐values are expressed as ^*^ *p *< 0.05, ^**^ *p* < 0.01, and ^***^ *p* < 0.001. ns, not significant.

## Conflict of Interest

The authors declare no conflict of interest.

## Supporting information



Supporting Information

## Data Availability

The data that support the findings of this study are available from the corresponding author upon reasonable request.
