# Supplementary material for: Targeted Delivery of c(RGDfk)‐Modified Liposomes to Bone Marrow Through In Vivo Hitchhiking Neutrophils for Multiple Myeloma Therapy
Source: Adv Sci (Weinh). 2024 Dec 16;12(12):2409895. doi: 10.1002/advs.202409895 (PMC11948077; doi:10.1002/advs.202409895)
Supplement: Supplementary file 1 — Supporting Information [file ADVS-12-2409895-s001.docx]

**Supplementary information**


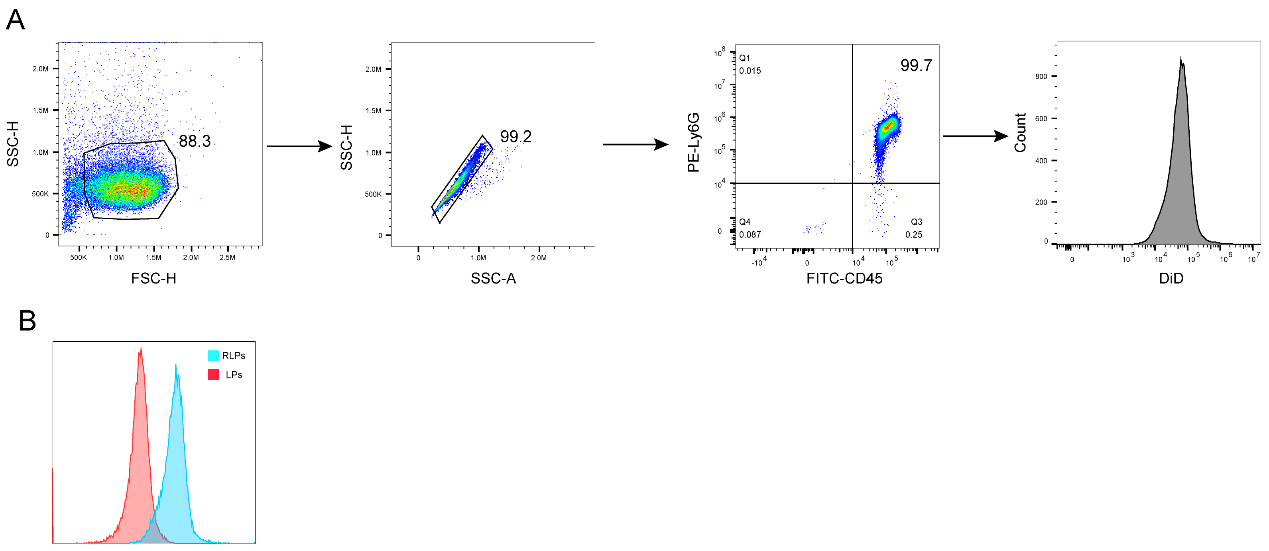


Fig. S1. The flow cytometry gating strategies (A) and representative results (B) for MFI of neutrophils after incubation with DiD-labeled LPs and RLPs for 1 h.


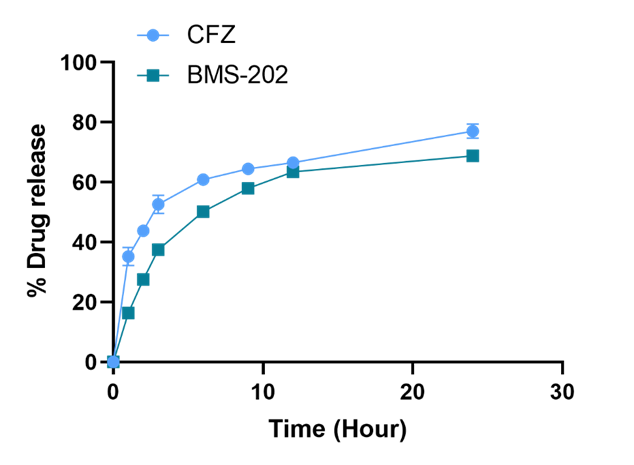


Fig. S2. The release curve of carfilzomib and BMS-202 from CRLPs and BRLPs in PBS containing 0.5% Tween 80 at pH 7.4, respectively. (n=3)


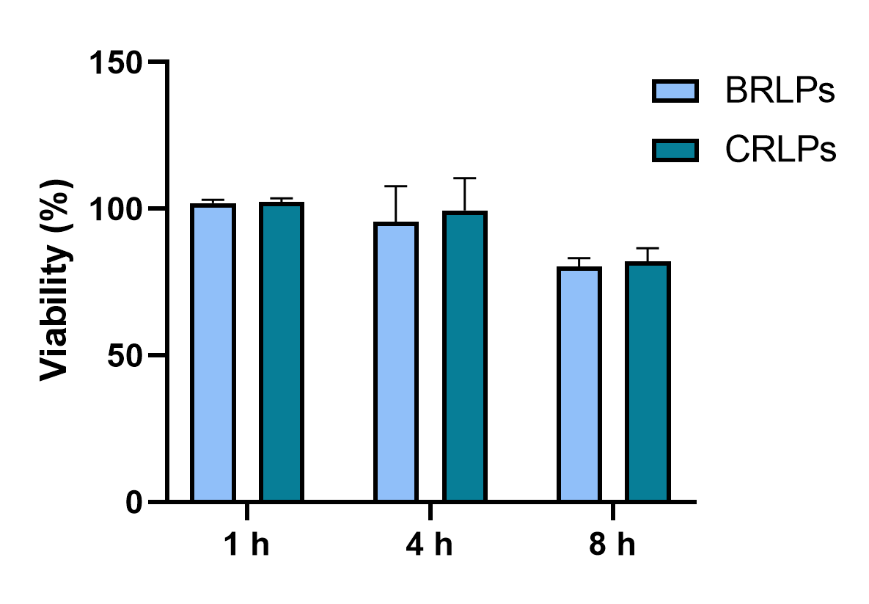


Fig. S3. The cell viability of neutrophils after incubated with BRLPs or CRLPs containing 120 nmol/L BMS-202 or CFZ for different time points (n=4).


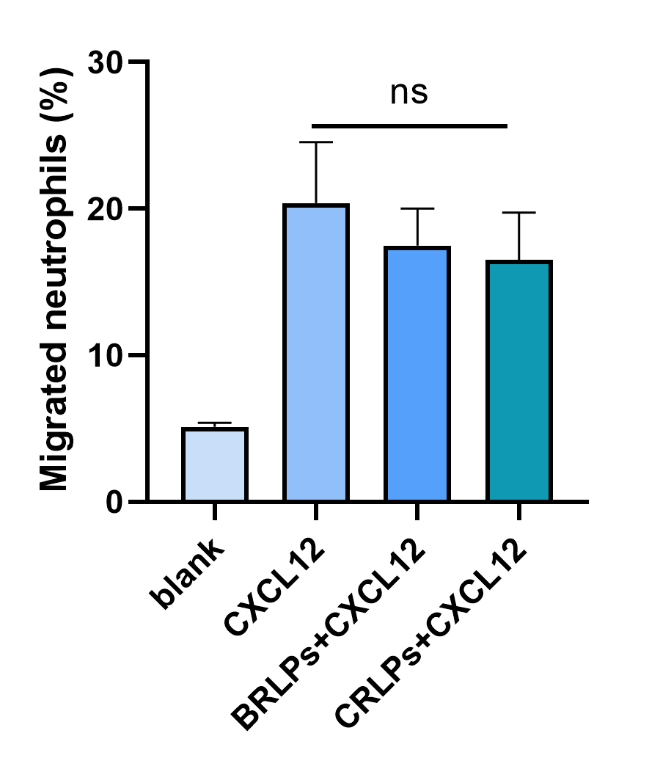


Fig. S4. The proportion of neutrophils that migrated to the lower chamber following treatment with BRLPs and CRLPs for 1 h in the CXCL12 chemotaxis transwell assay. The data were presented as mean ± SEM (n=4). The abbreviation “ns” indicated no significant difference.


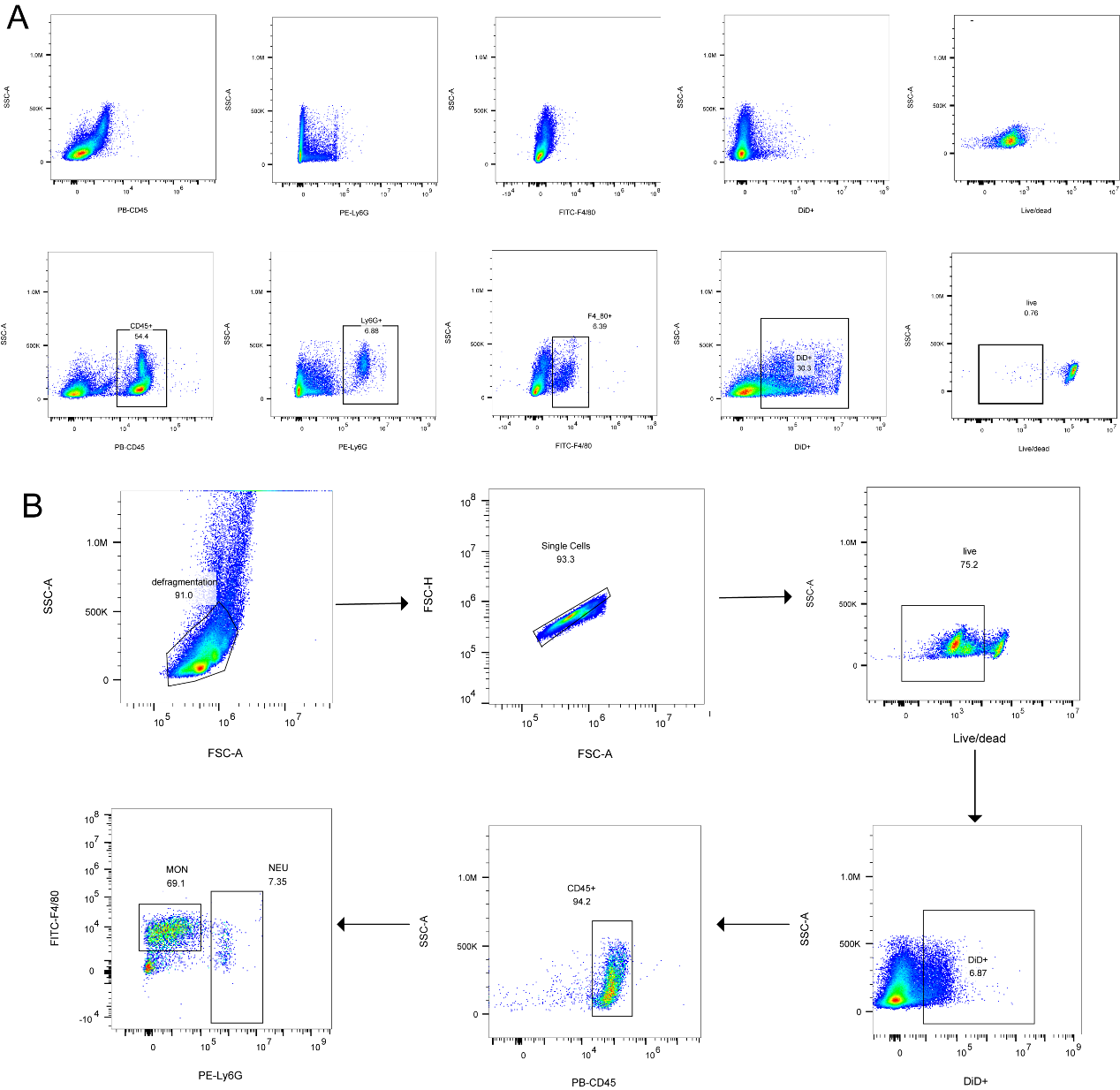


Fig. S5. The flow cytometry data for unstained and single-stained controls (A), as well as the representative gating plots for neutrophils and monocytes in peripheral blood (B).

Fig. S6. The mean fluorescence intensity (MFI) plot determined by flow cytometry illustrating CXCR4 expression on neutrophils after *in vitro* incubation with RLPs, BRLPs, and CRLPs for 1, 4, and 8 h. The data were presented as mean ± SEM (n=4). The abbreviation “ns” indicated no significant difference.


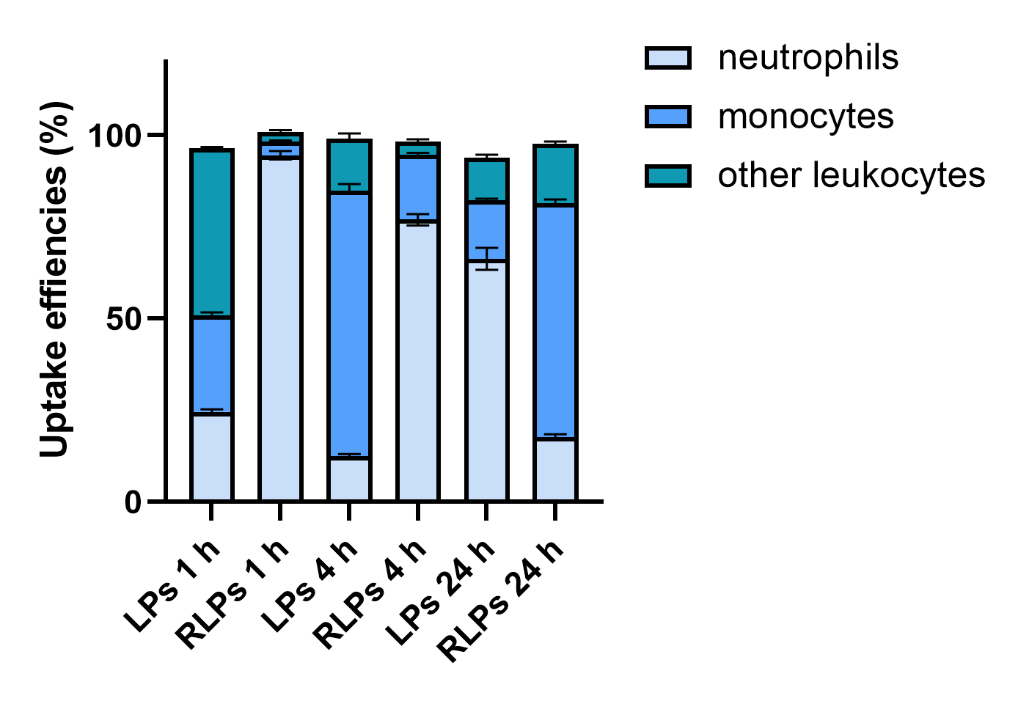


Fig. S7. The uptake efficiencies of LPs and RLPs in different types of leukocytes at various time points after administration with DiD-labeled LP or RLPs (n=3).


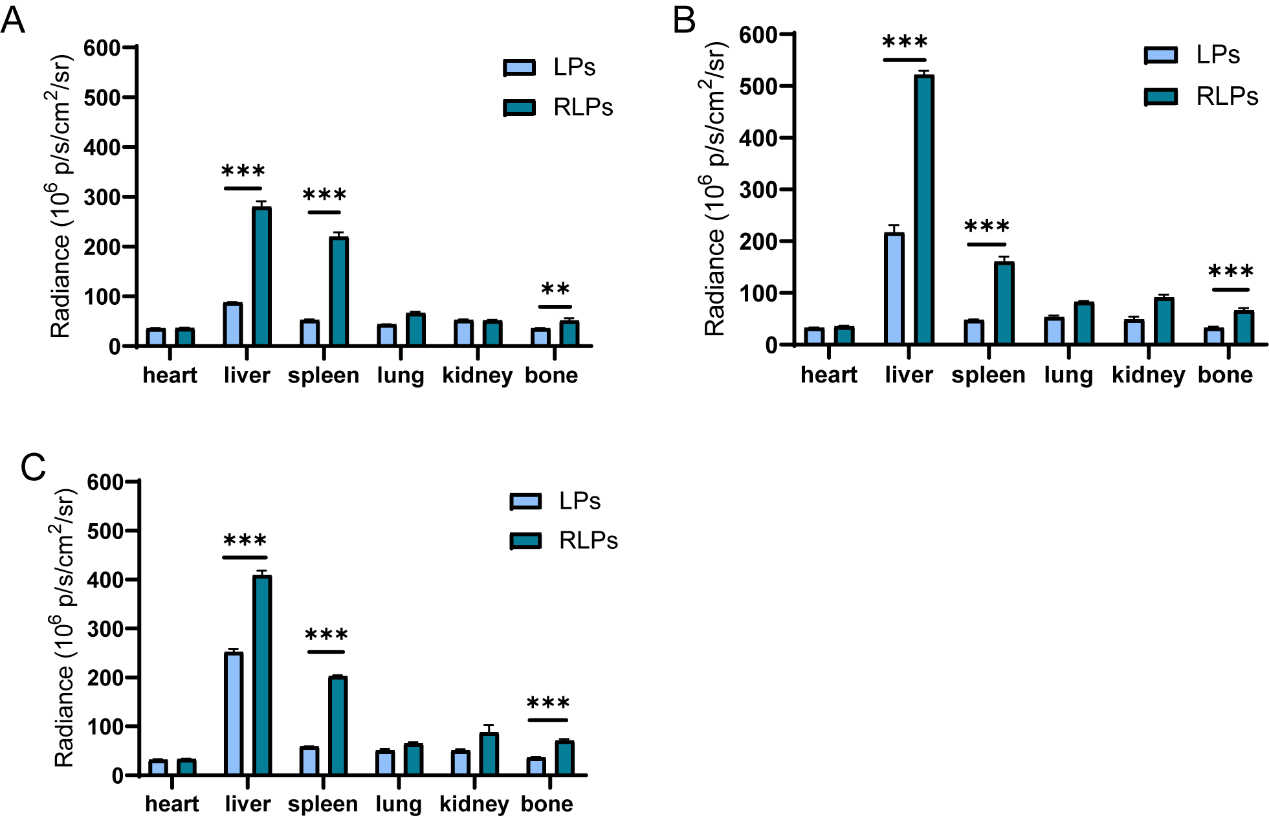


Fig. S8. (A-C) The biodistribution of DiD-LPs or DiD-RLPs in normal mice at 1h (A), 4h (B) and 24h (C) after intravenous injection of DiD-LPs or DiD-RLPs. The data were presented as mean ± SEM (n=3). ** *P* < 0.01, and *** *P* < 0.001.


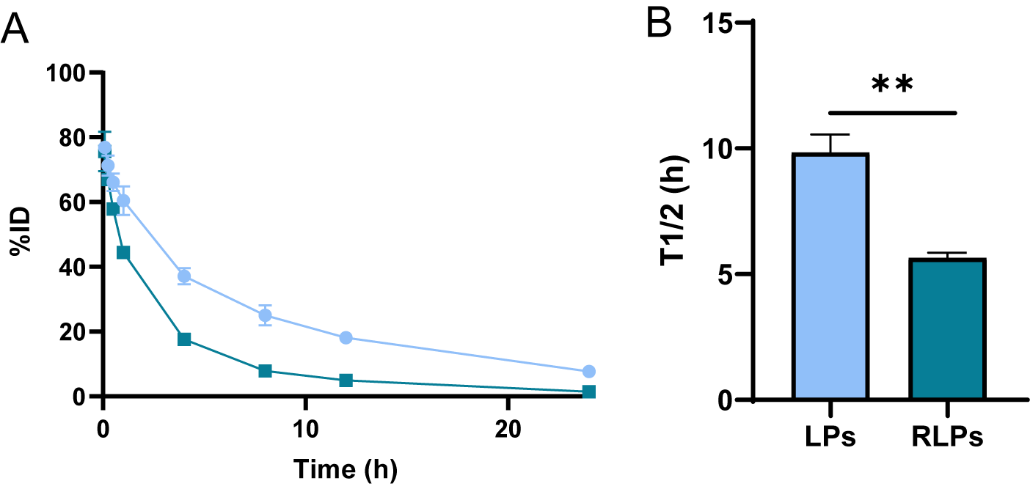


Fig. S9. The pharmacokinetic profiles of LPs and RLPs after intravenous injection. (A) The percentages of injected dose (%ID) were plotted against the time. (B) The circulation half-life of LPs or RLPs in normal mice following intravenous injection. The data were presented as mean ± SEM (n=3). ***P* < 0.01.


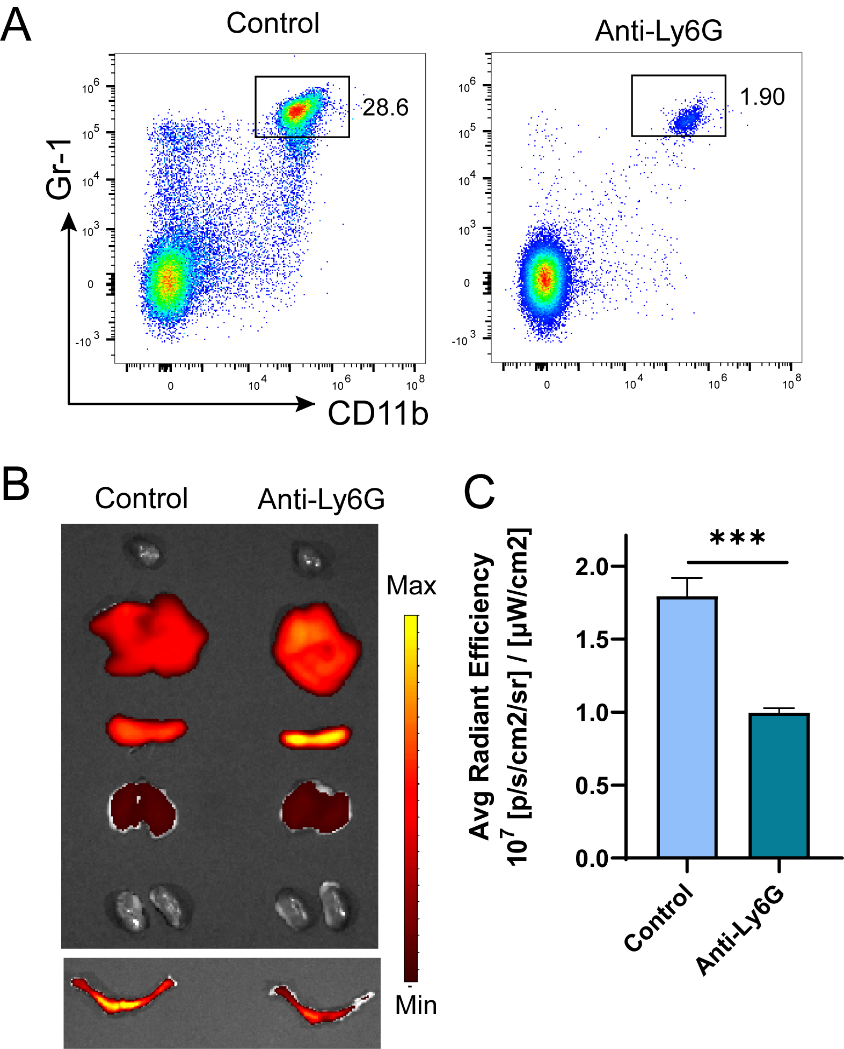


Fig. S10. The effect of neutrophil depletion on delivery of RLPs to bone marrow. (A) Representative plots of neutrophils in blood of control and anti-Ly6G-treated recipient mice. Neutrophils were indicated as CD11b^+^Gr-1^+^. (B, C) Representative fluorescence images of important organs (B) and the corresponding quantitative assessment of the average fluorescence intensity of femur and tibia at 4 h (C) following intravenous administration with DiD-RLPs. The data were presented as mean ± SEM (n=3). *** *P* < 0.001.


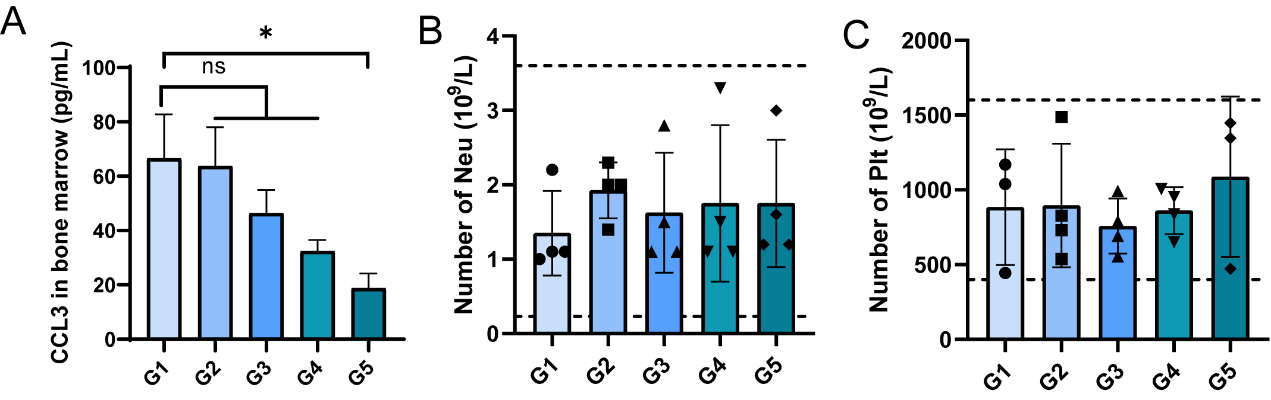


Fig. S11. The levels of CCL3 in mouse bone marrow at the pharmacodynamic endpoint (A), along with neutrophil counts (B) and platelet counts (C) in whole blood. G1, the PBS group. G2, the BLPs+CLPs group. G3, the BRLPs group. G4, the CRLPs group. G5, the BRLPs+CRLPs group.


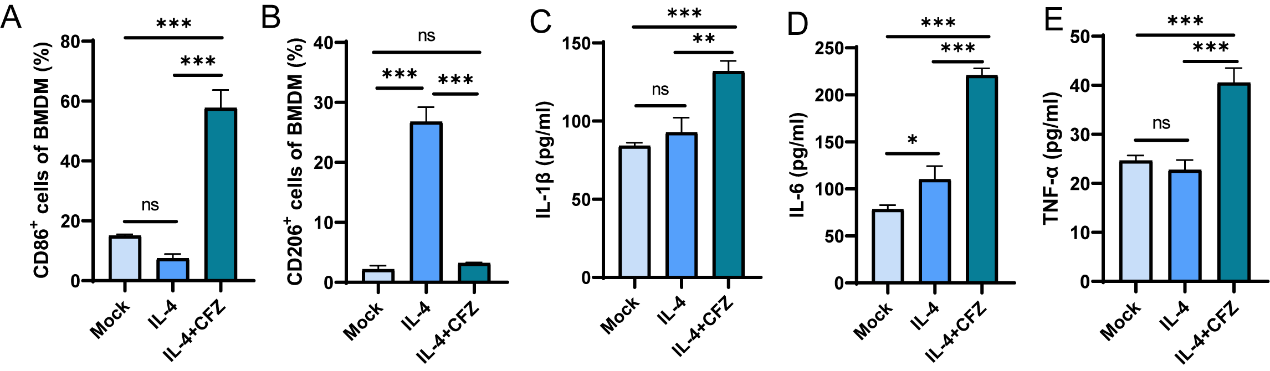


Fig. S12. (A-E) Carfilzomib induced a phenotypic shift of tumor-associated macrophages from an M2 to an M1 phenotype. (A, B) The flow cytometry results depicting the percentage of CD45^+^F4/80^+^CD86^+^ cells in bone marrow derived macrophages (BMDMs) (A) and the percentage of CD45^+^F4/80^+^CD206^+^ cells in BMDMs (B). (C-E) Proinflammatory cytokine secretion from BMDMs after different treatments. The data were presented as mean ± SEM (n=4). * *P* < 0.05, ** *P* < 0.01, and *** *P* < 0.001. The abbreviation “ns” indicated no significant difference.


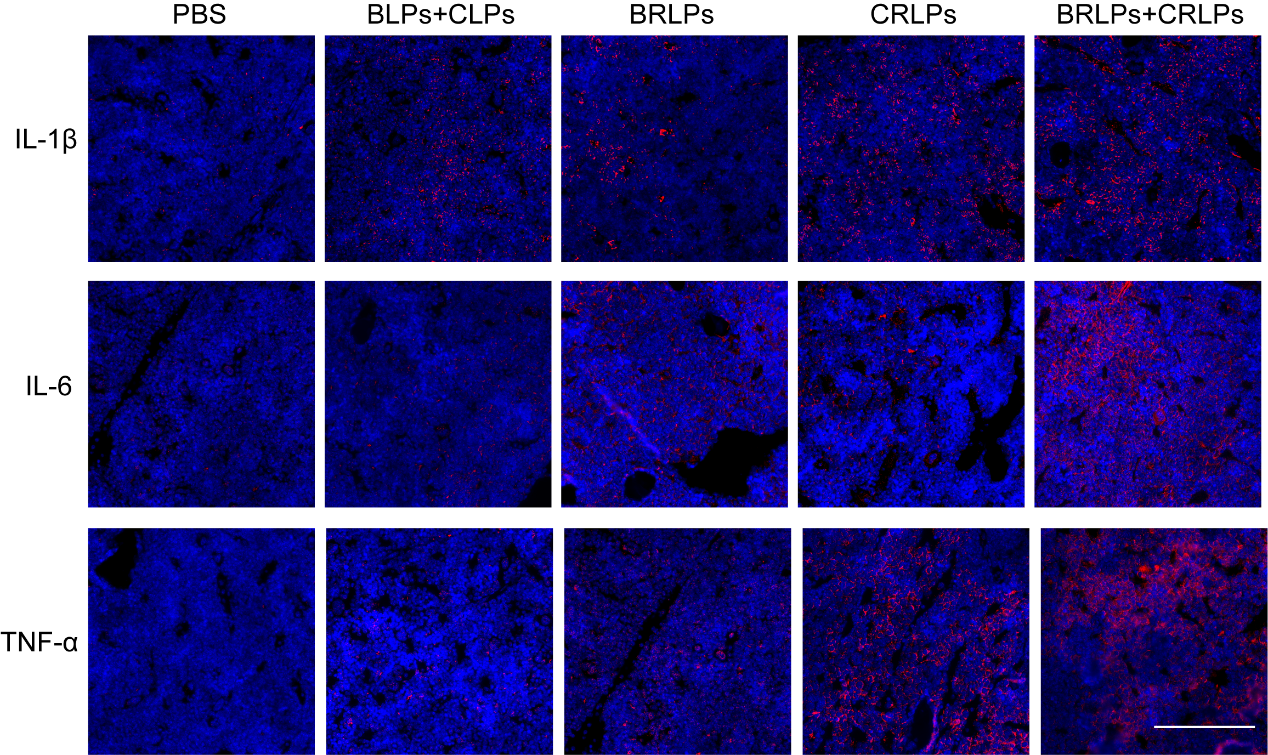


Fig. S13. Representative immunofluorescence images of proinflammatory cytokines including IL-1*β*, IL-6 and TNF-*α* in the bone marrow of MM mouse models after different treatments. Scale bar, 50 μm.


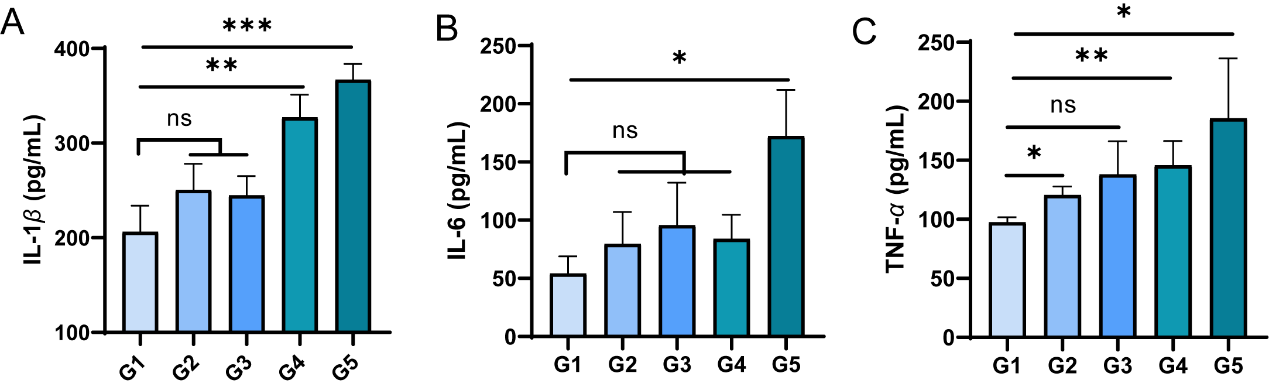


Fig. S14. The cytokine levels of IL-1*β* (A), IL-6 (B), and TNF-*α* (C) in mouse serum measured by ELISA. The data were presented as mean ± SEM (n=4). G1, the PBS group. G2, the BLPs+CLPs group. G3, the BRLPs group. G4, the CRLPs group. G5, the BRLPs+CRLPs group. * *P* < 0.05, ** *P* < 0.01, and *** *P* < 0.001. The abbreviation “ns” indicated no significant difference.


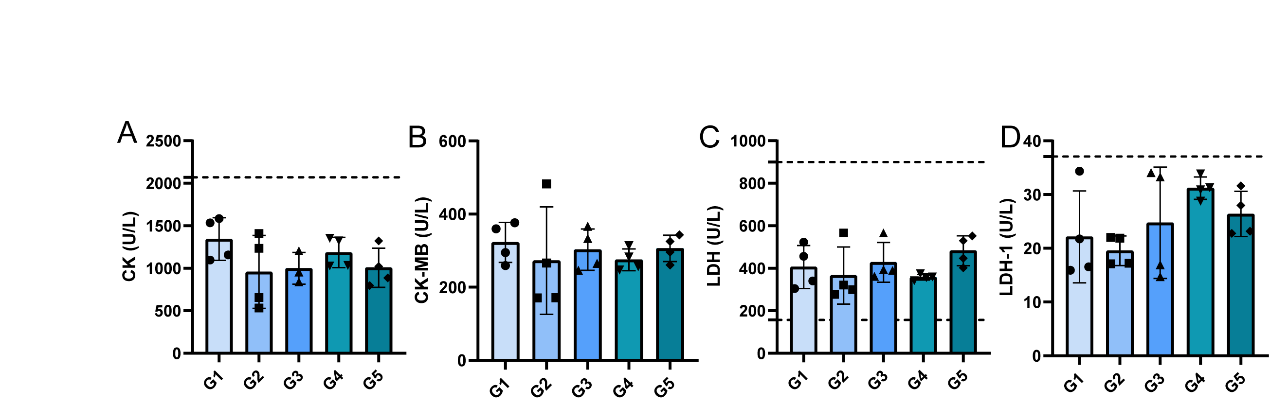


Fig. S15. The levels of serum myocardial enzymes, including CK, CK-MB, LDH, and LDH-1, from mice models after different drug treatments. The data were presented as mean ± SD (n=4).
